# Investigation of Radiation Damage in the Monazite-Type Solid Solution La_1–*x*_Ce_*x*_PO_4_

**DOI:** 10.1021/acs.inorgchem.4c02041

**Published:** 2024-09-04

**Authors:** Theresa Lender, Gabriel Murphy, Elena Bazarkina, Andrey Bukaemskiy, Sara Gilson, Maximilian Henkes, Christoph Hennig, Alexander Kaspor, Julien Marquardt, Jonas Nießen, Lars Peters, Jenna Poonoosamy, André Rossberg, Volodymyr Svitlyk, Kristina O. Kvashnina, Nina Huittinen

**Affiliations:** † Institute of Crystallography, RWTH Aachen University, Aachen 52066, Germany; ‡ Institute of Fusion Energy and Nuclear Waste Management (IFN-2), Forschungszentrum Jülich GmbH, Jülich 52428, Germany; § Institute of Resource Ecology, Helmholtz-Zentrum Dresden-Rossendorf, Dresden 01328, Germany; ∥ The Rossendorf Beamline at ESRF, The European Synchrotron, Grenoble 38043, France; ⊥ Institut für Geowissenschaften, Goethe-Universität Frankfurt, Frankfurt am Main 60438, Germany; # Institute of Mineral Engineering, RWTH Aachen University, Aachen 52074, Germany; ∇ Institute of Chemistry and Biochemistry, Freie Universität Berlin, Berlin 14195, Germany

## Abstract

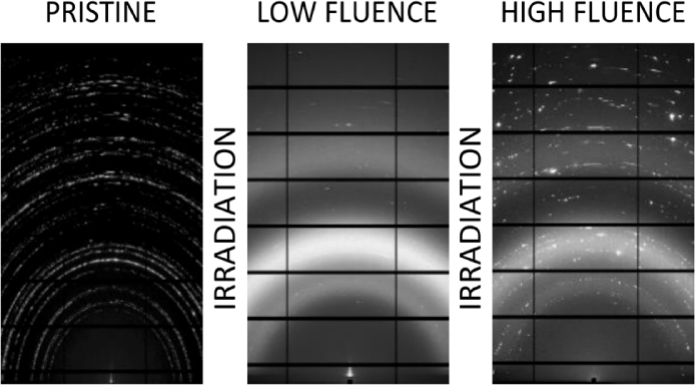

Crystalline materials such as monazite have been considered for the storage of radionuclides due to their favorable radiation stability. Understanding their structural chemical response to radiation damage as solid solutions is a key component of determining their suitability for radionuclide immobilization. Herein, high-resolution structural studies were performed on ceramics of the monazite solid solution La_1–*x*_Ce_*x*_PO_4_ (*x* = 0.25, 0.5, 0.75, 1) in order to understand the role of structural chemistry on irradiation stability. Ceramic samples were irradiated with 14 MeV Au ions with 10^14^ ions/cm^2^ and 10^15^ ions/cm^2^ to simulate the recoil of daughter nuclei from the alpha decay of actinide radionuclides. The extent of radiation damage was analyzed in detail using scanning electron microscopy (SEM), Raman spectroscopy, grazing incidence X-ray diffraction (GI-XRD), and high-energy-resolution fluorescence detection extended X-ray absorption fine structure (HERFD-EXAFS) spectroscopy. SEM and Raman spectroscopy revealed extensive structural damage as well as the importance of grain boundary regions, which appear to impede the propagation of defects. Both radiation-induced amorphization and recrystallization were studied by GI-XRD, highlighting the ability of monazite to remain crystalline at high fluences throughout the solid solution. Both, diffraction and HERFD-EXAFS experiments show that while atomic disorder is increased in irradiated samples compared to pristine ceramics, the short-range order was found to be largely preserved, facilitating recrystallization. However, the extent of recrystallization was found to be dependent on the solid solution composition. Particularly, the samples with uneven ratios of solute cations, La_0.75_Ce_0.25_PO_4_ and La_0.25_Ce_0.75_PO_4_ were observed to exhibit the least apparent radiation damage resistance. The findings of this work are discussed in the context of the monazite solid solution chemistry and their appropriateness for radionuclide immobilization.

## Abstract 2

The extraordinary recrystallisation ability of monazite throughout the La1-xCexPO4 solid solution is demonstrated in this study on various compositions irradiated with 14 MeV Au ions to simulate the effect of recoil nuclei from alpha decay. While at a fluence of 1014 ions/cm^2^, the samples are almost fully X-ray amorphous, a significant recovery of the crystalline structure is observed at 1015 ions/cm^2^ in all compositions.

## 1 Introduction

Monazite is an important natural source of rare earth elements^1,2^ and a major source of thorium.^3^ Monazite-type phosphates have a wide range of technical applications including catalysis, proton conduction, fluorescent labeling in cell biology, or as thermal barrier coatings.^4−7^ Due to its high chemical durability and the frequent occurrence of uranium in the monazite structure, it is commonly used for geochronological studies.^8,9^ These properties also substantiate the ongoing interest in monazite as a potential ceramic host for high-level nuclear waste.^10−12^ The particularly high chemical flexibility of monazite has been underlined by numerous studies that have shown perfect miscibility between the lanthanides La–Gd^13−15^ as well as successful incorporation of the actinides Pu, Am, *Cm*, Bk, Cf, and Es.^16−18^ Substitution of further elements like S, As, F and V has been reported among others by Aldred et al.^19^ and Ondreijka et al.^20^ The exceptional durability of monazite is evidenced by the presence of monazite in beach sands and placer deposits that have been produced by weathering of granitic host rocks.^21^

**Figure 1:**
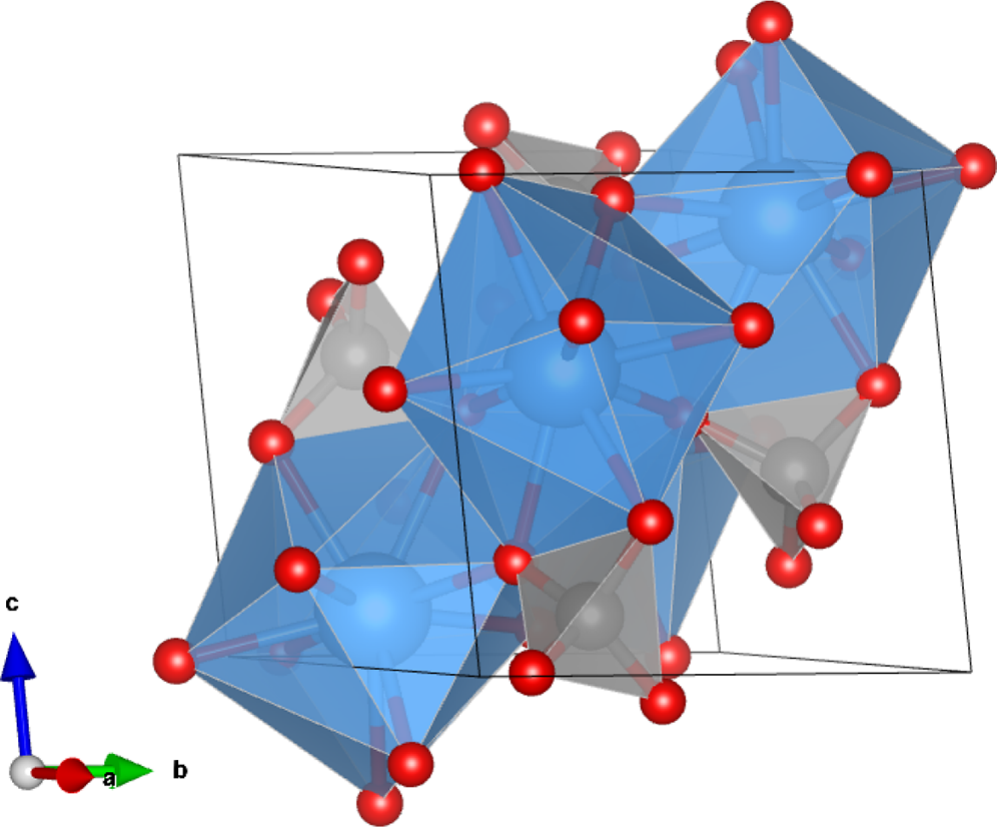
Monazite unit cell showing the framework consisting of *Ln*O_9_ polyhedra and PO_4_ tetrahedra.

The general formula for monazite-type compounds is *Ln*PO_4_ (*Ln* = La–Gd). It crystallizes in the monoclinic space group *P2*_*1*_*/n* (*Z* = 4). The structure is based on distorted *Ln*O_9_ polyhedra which are connected to PO_4_ tetrahedra in the [001] direction, the structure is generally described as a phosphate framework (see Figure 1).^22^ Every *Ln*O_9_ polyhedron shares one oxygen atom with five surrounding chains in the --plane, respectively. This has been called the locking effect.^23^

Resistance against radiation damage is one of the most fundamental requirements of host phases for the long-term storage of radioactive waste. Most of this damage is introduced by alpha decay of radionuclides generating alpha particles (He^2+^), recoil of the formed daughter nuclei, and, to some extent, by gamma emission.^24^ Alpha particles transfer 99.8% of their energy via ionization and electronic excitation processes and only introduce a few hundred atomic displacements.^25^ In contrast, alpha-recoil cascades contain 1000–2000 atomic displacements.^24^ This damage is known to introduce structural changes in crystalline materials such as swelling,^26,27^ decreased hardness,^25^ decreased thermal conductivity,^28^ and increased diffusion coefficients, both due to percolating clusters forming within the material and due to amorphization.^29−31^ It is therefore essential for possible waste matrices to show high resistance toward radiation damage as well as good recrystallization properties.

While it is possible to amorphize monazite under ion irradiation^32,33^ or by incorporation of potent alpha-emitters like^241^Am and ^238^Pu,^17,34,35^ there appears to be no report about natural metamict samples regardless of high levels of radionuclides commonly encountered in these samples. In literature this fact has been attributed to the exceptional damage recovery based on a combination of low critical temperatures (as low as 60 ^◦^C in synthetic LaPO_4_^36^) and the capacity for radiation-induced recovery.^37−39^ This remarkable stability is one of the main reasons why monazite is being considered as one of the most promising candidates as a high-level waste storage matrix. So far, studies have focused on the various monazite end members,^11,40−42^ although nuclear waste forms are likely to be far more complex in terms of elemental composition. Accordingly, understanding the structural–chemical response of monazite solid solutions is crucial to assessing their applicability in waste form application and actinide immobilization.

Irradiation with heavy ions makes it possible to attain considerable damage levels within short time frames with a high degree of control over the experimental conditions. Gold ions have been used before to simulate recoil atoms from alpha-decay^43^ even though the energy fraction transferred via inelastic processes is higher compared to recoil nuclei.^25^ The avoidance of radioactive species facilitates sample handling and subsequent analytical investigation of radiation damage. It must be considered though, that the stability of monazite against ion implantation has been shown to differ from the behavior observed in case of self-irradiation.^44^ The high dose rates applied in heavy ion irradiation generally overwhelm the thermal recovery taking place on longer time scales.^24^ On the other hand, irradiation has been shown to facilitate recombination of point defects in damaged areas.^39^ An important factor to consider is the material type for irradiation. In the context of monazite materials, ceramics are the likely option due to their ease of processing and generation, particularly compared to single crystals. Although single-phase ceramic materials should be homogeneous in terms of chemical composition, the inherent occurrence of variably sized grains and grain boundaries enhances the material heterogeneity. This can be further amplified by irradiation damage effects. This has been shown previously in a variety of compounds.^45−47^ Particularly, it has been shown that grain boundaries can act as sinks toward the accumulation of radiation defects in the case of zirconia materials.^48^ For monazite materials, similar observations have been made in CePO_4_.^40^ In such studies, radiation damage also appears to be linked to grain boundaries. Less clear is how radiation damage affects the material as a solid solution, i.e., a matrix closer to a real waste form with dissolved radioactive constituents within the structure, which contribute to solid solution mixing effects as seen for instance in pyrochlores among others. Indeed there is a relative paucity of information regarding the structural response of monazite solid solutions to radiation damage relevant to radionuclide disposal.

In an attempt to address this dearth of information and shed further light on the irradiation response of monazite solid solutions, ceramic samples with various compositions encompassing the solid solution La_1–*x*_Ce_*x*_PO_4_ (*x* = 0.25, 0.5, 0.75, and 1) were investigated with regard to their susceptibility to radiation damage. The samples were irradiated with 14 MeV Au ions at two different fluences: 10^14^ ions/cm^2^ (only CePO_4_ and La_0.5_Ce_0.5_PO_4_) and 10^15^ ions/cm^2^. While the former is slightly below the critical amorphization dose of CePO_4_ and should therefore not lead to full amorphization, the latter exceeds it and should result in the complete destruction of long-range order if no recrystallization occurs. Scanning electron microscopy (SEM) was used to assess topological effects of the irradiation. Raman measurements were performed to probe changes to local bonding environments and chemistry. Finally, the long- and short-range order of the irradiated compounds were probed by grazing-incidence X-ray diffraction (GI-XRD) and high energy resolution fluorescence detection extended X-ray fine structure (HERFD-EXAFS), respectively.

## 2 Methods

### 2.1 Samples

Precursor rhabdophane powders (*Ln*PO_4_ × 0.67 H_2_O) for both end members and solid solution compounds were prepared according to methods described in the literature.^49^ Targeted stoichiometric additions of cerium nitrate (99.99%, Sigma-Aldrich) and lanthanum nitrate (99.98%, Alfa Aesar) salts were dissolved in deionized water. Precipitation of rhabdophane was induced by the addition of phosphoric acid (85%, Sigma-Aldrich). The slurry solution was heated at 90 ^◦^C for approximately 12 h to allow full conversion to rhabdophane. The solid was separated and washed several times using deionized water with subsequent separation via centrifugation. For the final step, the solid was separated and suspended in 0.1 M HNO_3_ and left to sit overnight according the procedure of Babelot et al.^50^ The acid was then decanted off and the solid calcined for 3 h at 120 ^◦^C in air, prior to further calcination at 500 ^◦^C in air for 2 h to form monazite. The calcined products were ground to a fine powder by using an agate mortar and pestle. Thereafter, the calcined products were pressed into green pellets using an Oehlglass, Hahn and Kolb MP12 uniaxial cold press by applying a force of 38 kN (*p* = 450 MPa) to approximately 500 mg of material as reported previously.^50^ The obtained pellets were weighed geometrically to calculate the green densities. Pellets were then sintered in a tube furnace in air at 1400 °C for 5 h. Densities of the crystalline targets were calculated through geometric and hydrostatic weighing as dictated by Archimedean and modified Archimedean methods^50^ (for more information see Supporting Information, Density Measurements). As a final step, targets were polished using abrasive silicon carbide paper and 1 μm diamond paste on an automatic polishing table for approximately 15 min.

### 2.2 Irradiation

The ceramic pellets were mounted on a Si wafer where one half was covered with Al foil to protect it from irradiation (compare with Figure S1). The implantation chamber was evacuated to approximately 3 × 10^–7^ mbar and cooled to 77 K with liquid nitrogen to reduce the thermal load at the sample surface during irradiation. The samples were irradiated with 14 MeV Au ions with fluences of 10^14^ (CePO_4_ and La_0.5_Ce_0.5_PO_4_) and 10^15^ ions/cm^2^ (all samples). The irradiations were performed at the Ion Beam Center at the Helmholtz-Zentrum Dresden-Rossendorf, Germany (HZDR) using the 3 MV Tandetron Ion Implanter. The penetration depth of the Au ions into the monazite ceramics was calculated to be approximately 2 μm for all samples using the Stopping Range of Ions in Matter (SRIM) Monte Carlo simulation code^51^ (for more information see Supporting Inormation, Penetration Depth).

### 2.3 SEM and EDX

Microstructural and chemical characterization was carried out by electron microscopy (SEM) and energy dispersive spectroscopy (EDX) (FESEM Gemini 500, Zeiss, Oberkochen, Germany; EDX detector X-Max80, Oxford Instruments, Abingdon, Oxfordshire, UK). Due to subsequent surface-sensitive measurements, samples were not coated for the analyses. Operating at 1 kV acceleration yielded high-quality secondary electron images. However, employing the variable pressure mode to capture backscattered images with an acceleration voltage of 15 kV was essential. The increased chamber pressure allowed for charge compensation by gas molecules. Large-field EDX scans were used to determine the precise composition of the pellets.

### 2.4 Raman Spectroscopy

Raman spectroscopic mapping was performed using an automated inverted microscope, specifically a Witec alpha300 Ri Inverted Confocal Raman Microscope. The microscope is composed of an inverted base microscope, the Nikon Ti-2 U, equipped with a 70 mW Nd:YAG laser (λ = 532 nm), a thermoelectrically cooled charge-coupled device (CCD) and a 50x objective. For the measurements, the laser power was set to 30 mW and a grating of 600 grooves/mm was chosen.

Raman maps of 100 μm × 100 μm size were recorded with a setp size of 1 μm in the x-y direction and with an integration time of 0.5 s. The maps were analyzed using the Raman Tool Set software from Candeloro et al.^52^ with regard to the intensity and fwhm of the ν1 PO_4_ stretching band.

### 2.5 Grazing Incidence X-ray Diffraction

Diffraction experiments in grazing incidence (GI) mode were performed on the BM20 Rossendorf Beamline (ROBL, ESRF, Grenoble, France). For this, a special GI module developed at the Rossendorf beamline was used, which allows alignment of the sample surface with respect to the incoming beam. Details on the GI module and the corresponding alignment procedure can be found in Svitlyk et al.^53^ Data collection was performed at an incidence angle α = 1° chosen to achieve the desired X-ray penetration depth. The latter depends both on the chemical composition of the studied material and on the energy of the incident synchrotron radiation. For the monazite samples studied here, penetration depths as a function of α was calculated using the GIXA^54,55^ package which is based on X-ray scattering tables published in Henke et al.^56^ For the used energy of 12 keV (λ = 1.0332 Å), the corresponding X-ray penetration depth is equal to 0.4 μm (see Figure S3). This ensured that only irradiated material was probed. During data collection, samples were oscillated by 20° in order to improve diffraction averaging. Full 360° rotation was not possible since the studied pellets were half-irradiated and full rotation would result in the simultaneous measurement of pristine and irradiated parts.

The GI module was mounted on to the rotation axis of the heavy-duty XRD2 multipurpose diffractometer of ROBL.^57^ Calibration of the GI diffraction module with respect to the incoming beam and detector position was performed with a standard NIST 660c LaB_6_ powder, corresponding experimental procedure is described in Svitlyk et al.^53^ The size of the incoming synchrotron beam was set to 0.3 mm (horizontal) and 0.03 mm (vertical), and 2D data were recorded on a Pilatus 2 M detector. Data were subsequently reduced to 1D powder patterns with the Dioptas software^58^ and Fourier transformed using PDFgetX3.^59^ The 2θ range of all measurements was 1°–42.5° (λ = 1.0332 Å).

### 2.6 HERFD-XANES and HERFD-EXAFS

Ce L_3_-edge HERFD-XANES and HERFD-EXAFS measurements of the pristine and irradiated CePO_4_ and La_0.5_Ce_0.5_PO_4_ pellets in grazing incidence were performed at the ROBL beamline (BM20) of the ESRF in Grenoble, France.^57^ The storage ring was operated at 6 GeV with a ≈ 200 mA current in 7/8 + 1 filling mode. The incident photon energies were selected using a liquid nitrogen-cooled double Si(111) crystal monochromator, and higher harmonics were suppressed by two Si mirrors operating in total reflection mode. The vertically focused beam size was 50 × 2000 μm^2^. The incident energy was calibrated by using a standard crystalline powder of CeO_2_ pressed into a pellet; the Ce L_3_ edge energy position was set to 5723.2 eV as the maximum of the first derivative of the main-edge HERFD-XANES spectrum. Both the pristine (masked side) and the irradiated parts of the pellet were measured in fluorescence mode with a 9° incidence angle for HERFD-EXAFS measurements and 45° for HERFD-XANES. A Johann-type X-ray emission spectrometer in a vertical Rowland geometry available at BM20^60^ was equipped with five spherically bent crystal Ge(331) analyzers with a 1 m bending radius, and a silicon drift X-ray detector (©Ketek). For HERFD measurements at the Ce L_3_ edge (5723 eV), the spectrometer was aligned at the maximum of the Ce Lα1 emission line (4839.2 eV) using the (331) reflection and the 80.8° Bragg angle. Six to eight spectra were collected for each sample and then averaged. Data reduction and shell fits were conducted with EXAFSPAK.^61^ CePO_4_ and CeO_2_ were used as the Ce(III) and Ce(IV) standards for HERFD-XANES interpretation, respectively. In order to calculate the electron wave vector (k), the Ce L_3_-edge ionization potential was arbitrarily defined as *E*_0_ = 5723 eV. For shell fitting theoretical scattering phases and amplitudes were calculated using the ab initio code FEFF8.20^62^ based on a La-substituted CePO_4_ monazite structure. During fitting, scaling factor S0_2_ was fixed to 0.9.

An impediment for the analysis of the mixed composition is the small energy difference between the La L_2_ (5891 eV) and Ce L_3_-edges (5723 eV), reducing the interpretable EXAFS region to approximately k = 6 Å^–1^. In the case of the short k-range spectra the fitted EXAFS parameter like radial distances (R), coordination numbers (CN) and Debye–Waller factors (DW) correlate strongly with the shift in energy threshold (Δ*E*_0_) which is unknown a-priory. Thus, in order to avoid unreliable structural parameter, shell fits were performed on the CePO_4_ end member first in the complete EXAFS range (up to 10 Å^–1^) to determine the shift in energy threshold Δ*E*_0_, which was then fixed for all fits using a common, shorter k-range of 2–6 Å^–1^.

Based on the crystal structure of monazite, only the *Ln*-O shell and a *Ln*-P shell with 4 P atoms at an average distance of 3.6 Å were considered in the shell fit. The coordination number of these shells had to be fixed during fitting, as it is strongly correlated to the Debye–Waller factors.

## 3 Results

### 3.1 SEM and EDX

**Figure 2:**
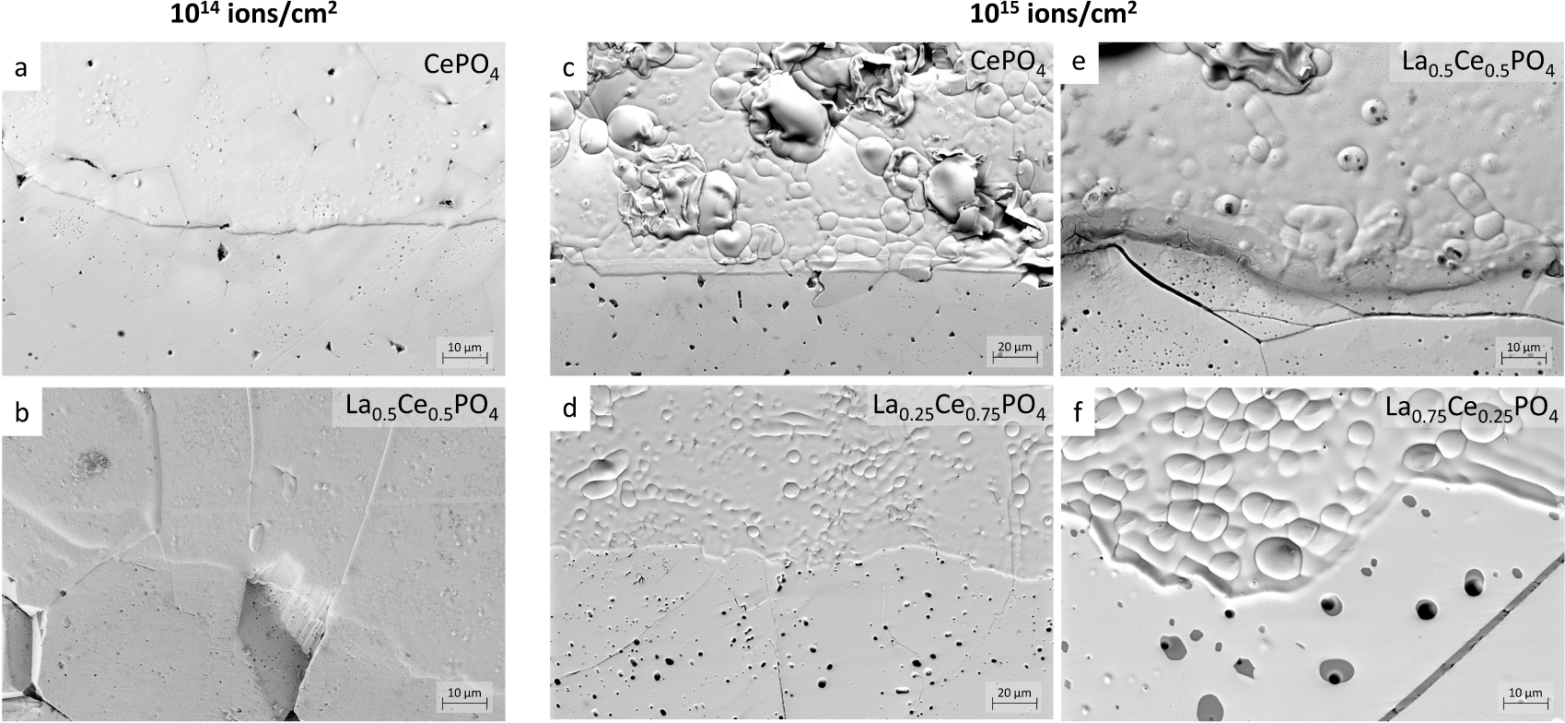
SEM images of the boundary between the irradiated (top) and pristine pellet (bottom). CePO_4_ and La_0.5_Ce_0.5_PO_4_ irradiated with 10^14^ ions/cm^2^ (a, b) and 10^15^ ions/cm^2^, respectively (c, d) as well as La_0.25_Ce_0.75_PO_4_ and La_0.75_Ce_0.25_PO_4_ irradiated with 10^15^ ions/cm^2^ (e, f). Radiation damage is clearly visible in all cases due to swelling of the crystallites, highlighting the grain boundaries. The strongest effect is visible in CePO_4_. Further images are shown in the SI (S4–S9).

SEM images were recorded at the interface between the irradiated and masked areas of the pellets to visually assess irradiation-induced changes in the sample topography. As shown in Figure 2, the interface between the pristine and irradiated part is clearly visible in all samples, indicated by swelling of the damaged region. This effect is more pronounced in samples irradiated with the higher fluence as well as strongly accentuated and rounded grain boundaries that might result from local melting or from swelling. The strongest topographical effect is observed in CePO_4_. In this case, individual grains seem to have swollen to the point of bursting. This indicates that the structural response of the end member toward irradiation is more severe than that of the solid solution members. As SRIM calculations do not show significant differences in structural damage introduced into the samples (see Supporting Information for detailed information), it may be concluded that the observed variations between the samples are based on various degrees of recrystallization. It is apparent that the grain boundaries play an important role in defect migration, as swelling is most pronounced at the centers of crystallites. EDS measurements confirmed the expected compositions (for more information, see Supporting Information).

### 3.2 Raman Spectroscopy

**Figure 3:**
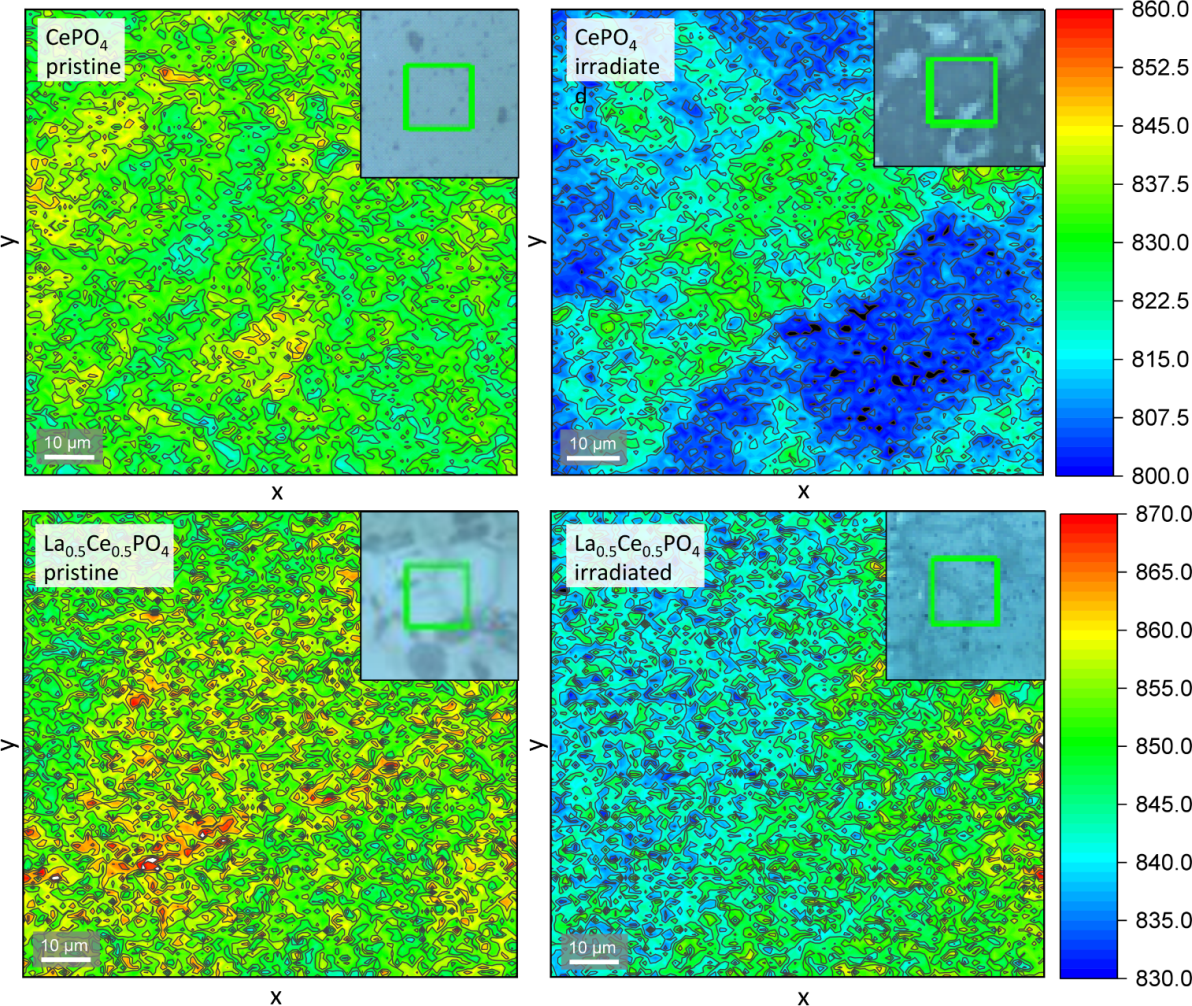
2-dimensional mappings of the intensity of the ν_1_ PO_4_ stretching mode measured by confocal Raman spectroscopy, shown for CePO_4_ and La_0.5_Ce_0.5_PO_4_ irradiated with 10^15^ ions/cm^2^. Lower intensities in the irradiated areas indicate the distortion of PO_4_ tetrahedra due to radiation damage. Intensity differences in irradiated samples appear to be linked to the individual grains. Mappings of all compositions can be found in the SI (Figure S10).

Raman maps were generated in both the pristine and irradiated areas of each pellet and analyzed to identify spectral differences that point toward radiation damage. Ruschel et al.^63^ recommended the fwhm of the ν_1_ PO_4_ stretching mode as the most suitable spectral parameter for the estimation of radiation damage in monazite since disorder commonly results in band-broadening and Raman stretching modes are particularly sensitive to disorder of neighboring atoms. However, no significant differences could be discerned with respect to this feature between the pristine and irradiated areas. By way of contrast, the intensity of the ν_1_ mode is significantly lower in damaged areas compared to undamaged material as shown in Figure 3. These results are particularly notable, since Nasdala et al.^64^ argued that confocal Raman spectrometers do not have a sufficient depth resolution in order to probe only the damaged surface area. They concluded that thorough analysis is possible only using thin lamellae. Since the peak height is dependent on the number of PO_4_ tetrahedra, conclusions can still be drawn about the degree of radiation damage using confocal Raman spectroscopy based on the relative change of intensity of the ν_1_ stretching mode without the necessity of preparing FIB lamellae.

The lack of band broadening can be traced back to the high degree of damage introduced by the high fluences used in this study. Nasdala et al.^64^ showed that at a fluence of 5.1 * 10^14^ ions/cm^2^ no significant Raman signal was obtained from the monazite lamella. Thus, at least in the case of the samples irradiated with 10^15^ ions/cm^2^ the irradiated layer is damaged to such an extent that it does not contribute to the Raman spectrum.

Upon closer inspection of the Raman maps, it becomes apparent that the range of intensities observed in the irradiated sections is larger than in the pristine areas, indicating regions with different levels of damage and that significant differences occur between individual grains. This suggests that grain boundaries act as barriers to the propagation of lattice defects. The ability of grain boundaries to act as defect sinks has been established before^65^ and becomes apparent again in these measurements.

Comparisons between different samples to assess the severity of the irradiation damage are not possible as the peak intensities depend on various factors, including crystallite orientation. Therefore, the absolute intensities between samples vary dramatically, irrespective of the degree of structural damage.

### 3.3 Grazing Incidence X-ray Diffraction

**Figure 4:**
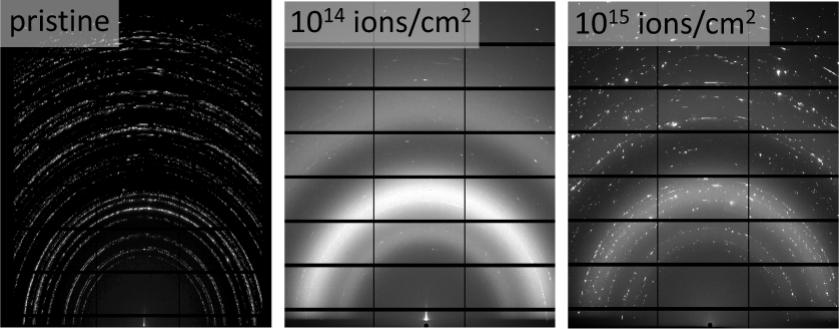
Diffraction images of a CePO_4_ pellet, left: pristine, middle: irradiated with 10^14^ ions/cm^2^, right: irradiated with 10^15^ ions/cm^2^. At 10^14^ ions/cm^2^ the sample is almost completely amorphous. Single crystal reflections are observed at 10^15^ ions/cm^2^ indicating a high degree of recrystallization.

Grazing incidence X-ray diffraction experiments were carried out to determine the level to which long-range order and crystallinity were preserved or regained in the damaged layer. The low incidence angles allow probing depths in the micrometer range, eliminating the influence of the underlying, undamaged bulk material. Detector images of a pristine CePO_4_ pellet as well as pellets irradiated with both fluences measured with an incidence angle α of 1° are shown in Figure 4. All of the pristine samples were fully crystalline. Due to the large crystallite sizes in the ceramics, their diffraction images exhibit single crystal diffraction spots in addition to Debye–Scherrer rings. The CePO_4_ pellet irradiated with 10^14^ ions/cm^2^ is almost completely amorphized with three broad amorphous rings being visible and very few crystalline reflections, which results in a very low crystallinity index of 12% (see Table 1). Debye–Scherrer rings at the same distances are also visible in the pellet irradiated with 10^15^ ions/cm^2^, but the intensity is significantly lower, and broadened single crystal reflections can be observed on top of the amorphous rings. These show strong variations in intensity indicating a broader distribution of crystallite sizes. The crystallinity index was determined to be 21%, considerably exceeding the crystallinity of the lower fluence sample.

**Table 1:** Crystallinity Index of Four Different Compositions Irradiated with Two Fluences

	fluence	
composition	10^14^ ions/cm^2^	10^15^ ions/cm^2^
CePO_4_	12%	39%
La_0.25_Ce_0.75_PO_4_		21%
La_0.5_Ce_0.5_PO_4_	6%	30%
La_0.75_Ce_0.25_PO_4_		22%

**Figure 5:**
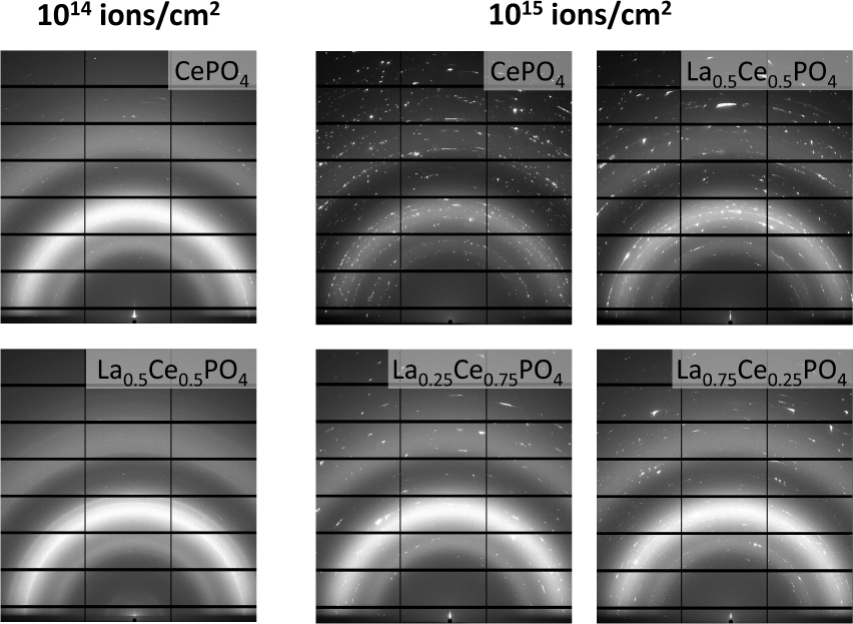
Diffraction images of CePO_4_ and La_0.5_Ce_0.5_PO_4_ (left top and bottom) irradiated with 10^14^ ions/cm^2^ and CePO_4_, La_0.25_Ce_0.75_PO_4_, La_0.5_Ce_0.5_PO_4_, and La_0.75_Ce_0.25_PO_4_ (middle top and bottom and right top and bottom) irradiated with 10^15^ ions/cm^2^, respectively. The damage is most severe in both samples irradiated at 1 × 10^14^ ions/cm^2^. The highest degree of crystallinity is observed in CePO_4_ at 10^15^ ions/cm^2^.

Similarly, La_0.5_Ce_0.5_PO_4_ exhibits a higher degree of damage at the intermediate fluence than at the highest fluence with crystallinity indices of 6 and 30%, respectively. These results underline the extent to which self-annealing of the monazite structure is possible given a sufficiently high energy input. Short local temperature spikes have been reported in ion-irradiated samples that enable annealing of structural defects.^66^ These, of course, depend on the fluence. With a fluence of 10^14^ ions/cm^2^ no evidence of recrystallization of the introduced damage is observable. In contrast, with 10^15^ ions/cm^2^ enough energy is introduced for damaged areas to recrystallize, resulting in the growth of large crystallites as evidenced by the bright spots in the diffraction images shown in Figure 5. In literature, dose rate effects have already been described with respect to damage generation in monazite.^11,67^ Based on our observations, we propose the same for self-annealing.

Notably, the two compositions La_0.25_Ce_0.75_PO_4_ and La_0.75_Ce_0.25_PO_4_ are more severely damaged with 10^15^ ions/cm^2^ than CePO_4_ and La_0.5_Ce_0.5_PO_4_ with crystallinity indices of 21% and 22%, respectively. One might expect CePO_4_ to recrystallize more readily considering the higher degree of order inherent to the end member structure. However, this does not explain the differing behavior between the La_0.5_Ce_0.5_PO_4_ on the one side and La_0.25_Ce_0.75_PO_4_ and La_0.75_Ce_0.25_PO_4_ on the other hand. This might be indicative of a possible asymmetry in the mixing enthalpy of the solid solution, as observed in La_1–*x*_Nd_*x*_PO_4_^68^ and La_1–*x*_Pr_*x*_PO_4_^69^ as well as pyrochlores,^70^ possibly resulting in a lower radiation resistance of these compositions. It cannot be excluded, though, that the differences arise from the fact that the different samples were obtained from independent synthesis runs. This is supported both by our SRIM calculations and by the study by from Picot et al.^25^ who did not see any significant differences between the susceptibility of LaPO_4_ and La_0.73_Ce_0.27_PO_4_ toward radiation damage. However, Burakov et al.^35^ describe clear differences between (La,Pu)PO_4_ and PuPO_4_ with respect to their resistance to self-irradiation. Indeed, a more pronounced effect might be observable in solid solutions containing cations with stronger variations of the ionic radius than La (1.216 Å) and Ce (1.196 Å), e.g., La_1–*x*_Gd_*x*_PO_4_.

**Figure 6:**
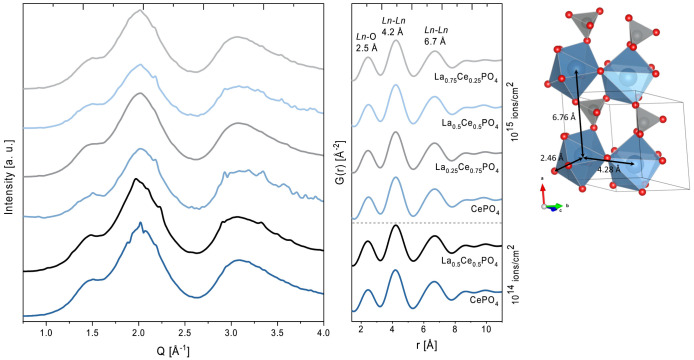
Left: Scattering of the amorphous fraction of irradiated pellets occurred in the Q-range up to 4 Å^–1^. Right: The dominant interatomic distances in the amorphous fraction correspond to the indicated atomic distances in the monazite structure on the right.

After carefully masking the Bragg reflections, the amorphous rings were integrated and smoothed to allow for a more detailed analysis of the amorphized material. All spectra show three broad signals at 1.5 Å^–1^, 2 Å^–1^, and 3 Å^–1^, as shown in Figure 6 on the left. Additionally, small features can be observed in CePO_4_ and La_0.5_Ce_0.5_PO_4_ irradiated at both fluences. These indicate that a higher degree of short-range order is retained than that in the other two solid solution samples.

Information about the atomic distances can be gained from the Fourier transforms of these spectra. Due to the low Q-range inherent to GI-XRD data, which is measured at low energies to reduce the penetration depth, no full analysis of the pair distribution function (PDF) was possible. Nevertheless, the same three dominant distances were identified in all irradiated samples as shown in Figure 6 on the right. These distances match closely some atomic distances found in the monazite structure: the average Ce–O distance in the CeO_9_ polyhedron (2.5 Å), the average of the Ce–Ce distance between individual LnO_9_–PO_4_ chains (4.2 Å), and the Ce–Ce distance corresponding to the unit cell parameter in the -direction (6.7 Å), respectively (compare Table 2). This shows that although the long-range order is lost due to the irradiation damage, the short-range order is largely retained. Especially, the preservation of LnO_9_ polyhedra can be observed. Additionally, a high degree of stability along the -direction can be inferred.

**Table 2:** Atomic distances Extracted from Amorphised Material Compared to those in CePO_4_ as Published by Bevara et al^71^

atomic distance	irradiated	pristine
Ce–O	2.45 Å	2.446 Å - 2.767 Å
Ce–Ce along [1 3/8 1]	4.21 Å	4.2874(5) Å
Ce–Ce along [1 0 0]	6.69 Å	6.7657(7) Å

The single crystal reflections visible in Figure 5 for CePO_4_ irradiated with 10^15^ ions/cm^2^ were integrated and compared to monazite diffraction patterns. The peak positions were found to coincide, verifying that the recrystallized phase is indeed monazite.

### 3.4 HERFD-XANES and HERFD-EXAFS

HERFD-XANES measurements on the Ce L_3_-edge were carried out to investigate a possible change in oxidation state of cerium due to the irradiation.^72,73^ No such change was observed and cerium was trivalent in all samples.^74^ A broadening of the white line can be attributed to the partial amorphization of the sample. The spectra are shown in Figure S12.

**Figure 7:**
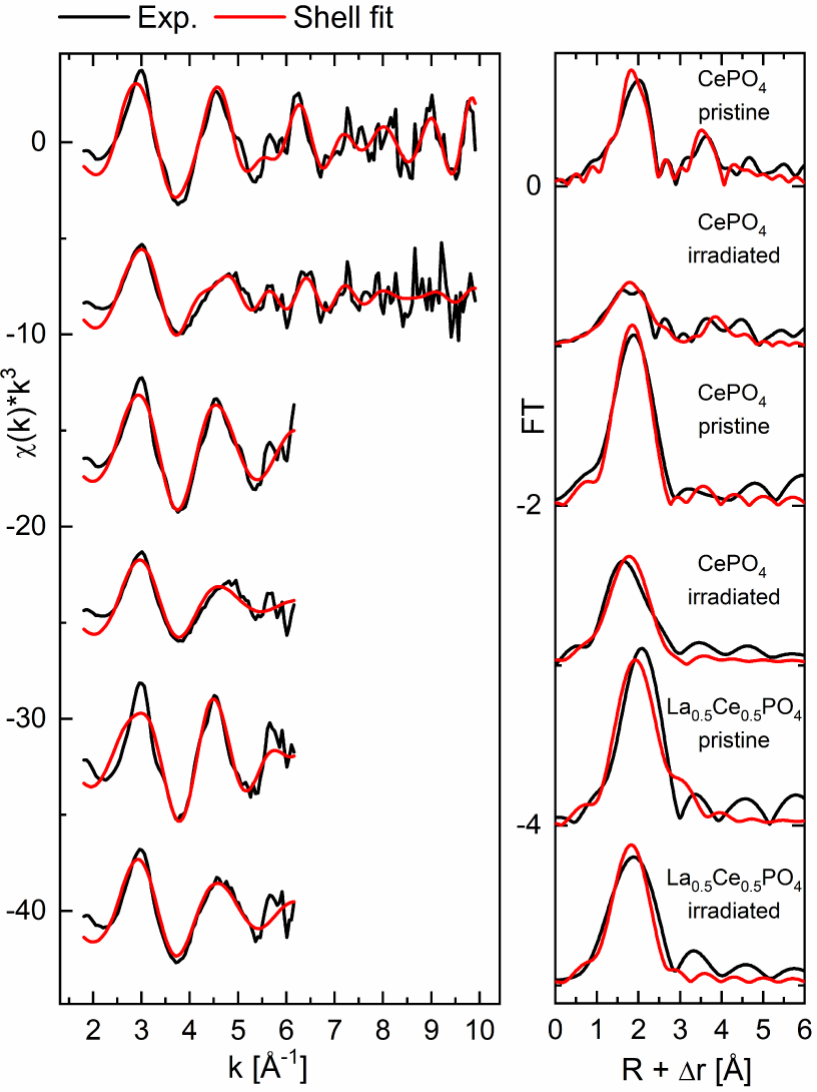
Shell fits (red) of the HERFD-EXAFS measurements (black) of pristine and irradiated CePO_4_ and La_0.5_Ce_0.5_PO_4_.

HERFD-EXAFS measurements were performed to study changes in the short-range order arising from irradiation. The compositions CePO_4_ and La_0.5_Ce_0.5_PO_4_ were analyzed both in the pristine state and after irradiation with 10^14^ ions/cm^2^. To increase comparability, both compositions were then analyzed in the short-range up to 6 Å^–1^ available for the mixed sample. The fits are shown in Figure 7, and the fit parameters are summarized in Table 3. Details regarding the fitting strategy and the fixed and fitted parameters are given in the Methods section.

**Table 3:** Shell Fit EXAFS Structural Parameters for CePO_4_ and La_0.5_Ce_0.5_PO_4_^a^

path	CN	R [Å]	DW [Å^2^]	Δ*E*_0_ [eV]
CePO_4_ pristine, full range				
O	9f	2.472(9)	0.026(1)	6.9(3)
P	4f	3.76(3)	0.023(5)	6.9/
Ce1	3f	4.023(7)	0.0016(9)	6.9/
Ce2	3f	4.216(8)	0.0016/	6.9/
CePO_4_ irradiated, full range				
O	9f	2.43(1)	0.049(3)	6.9f
P	4f	3.70(3)	0.029(6)	6.9/
Ce1	3f	4.01(2)	0.014(2)	6.9/
Ce2	3f	4.56(2)	0.014/	6.9/
CePO_4_ pristine, short range				
O	9f	2.464(5)	0.027(1)	6.9f
P	4f	3.68(7)	0.06(2)	6.9/
CePO_4_ irradiated, short range				
O	9f	2.426(9)	0.051(3)	6.9f
P	4f	3.72(9)	0.07(3)	6.9/
La_0.5_Ce_0.5_PO_4_ pristine				
O	9f	2.484(7)	0.029(2)	6.9f
P	4f	3.53(2)	0.014(3)	6.9/
La_0.5_Ce_0.5_PO_4_ irradiated				
O	9f	2.454(7)	0.038(2)	6.9f
P	4f	3.80(7)	0.06(2)	6.9/

a CN = coordination number, R = radial distance, DW = Debye-Waller factor, ΔE_0_ = shift in energy threshold. Fixed parameters are marked with f, and linked parameters with /. Standard deviations as estimated by EXAFSPAK are given in parentheses.

Strong peak broadening in the irradiated samples becomes immediately apparent, as indicated by the increased Debye–Waller factors. This indicates a higher degree of disorder in the local environment due to irradiation-induced defects. Additionally, the average *Ln*-O distances decrease during irradiation, which corroborates the results obtained from the PDF analysis, Table 2. The *Ln*-P distances increase when the shorter k-range is considered. For the full range, however, the distance appears to be very similar, especially when considering the rather large error associated with this *Ln*-P distance. Thereby, definite conclusions about this second scattering shell cannot be drawn from the fitted data.

A possible explanation for the decreasing *Ln*-O bond distance can be found in the SRIM calculations. The vast majority of displacements introduced by ion bombardment are oxygen vacancies, while phosphorus atoms are significantly less likely to be displaced. Thus, the latter bond distances are less affected by the irradiation, or they even become slightly longer, as expected in a distorted structure, while the former may be separated into a minority of displaced atoms with very long *Ln*-O distances and a majority of oxygen atoms experiencing a trend toward 8-fold coordination. A very asymmetric *Ln*-O bond length distribution in the irradiated materials would also explain the FT’s of the CePO_4_ sample (Figure 7, right), where the first scatting shell shows an apparent splitting (full range fit) or a clear asymmetry (short-range fit) following irradiation. A radiation-induced reduction of oxygen coordination has been observed before in perovskites^75^ and zirconolites.^76^ The Ce peak at R+Δr = 3.5 Å, which is clearly visible in pristine CePO_4_, has almost disappeared in the irradiated CePO_4_ sample. The absence of the Ce peak indicates larger differences among the Ce–Ce distances, which result in destructive interferences of the corresponding backscattering contributions. This effect is well-known for highly disordered structures.^77^

A further pertinent observation from the present investigation is the reduced radiation resistance of the solid solution members La_0.25_Ce_0.75_PO_4_ and La_0.75_Ce_0.25_PO_4_ compared to the end and middle member under the fluence 10^15^ ions/cm^2^ (Figure 5). Simultaneously, these two compositions under the same fluence appear to exhibit a relatively minimized swelling (Figure 2). This variability and nonlinearity of structural behavior in the solid solutions from irradiation damage has been previously noted in titanate perovskite solid solutions. Smith et al.^78^ pointed to the significance of defect-assisted recovery and enhanced amorphization from ion-irradiation depending on composition and position of the solid solution.^78^ The salience of defect processes in radiation damaged solid solutions has also been highlighted in pyrochlore oxides, where Lumpkin et al.^79^ highlighted the significant role of cation/anion energetics in inducing disorder. With the present data and lack of simulation methods applied, it is difficult to draw definitive conclusions in the present case for the apparent reduced radiation resistance and minimized swelling of La_0.25_Ce_0.75_PO_4_ and La_0.75_Ce_0.25_PO_4_ compared with other studied members. However, it is postulated that the uneven configuration of La and Ce cations in these samples (compared to LaPO_4_ and La_0.5_Ce_0.5_PO_4_) and particularly the susceptibilities to displacement of different individual cations from ion irradiation are linked.^80^ Grain boundaries have been previously described as sinks toward the migration and accumulation of radiation defects and potential recrystallization.^48^ Any heterogeneity of this process, particularly variability in the accumulation of unlike cations (La and Ce) due to their different susceptibilities to displacement from ion irradiation, can potentially impact recrystallization and in turn may reduce swelling. This description lacks a theoretical basis to properly describe it. Nevertheless, it is apparent from this study that “uneven” solid solutions in which the solution cations are not present in equal quantities appear to reduce the radiation tolerance of monazite materials. This accordingly may impact the design and application of such or similar materials for actinide immobilization and waste form purposes.

## 4 Conclusion

Ceramic pellets of different compositions of the La_1–*x*_Ce_*x*_PO_4_ solid solution (*x* = 0.25, 0.5, 0.75, and 1) were irradiated with 14 MeV Au ions using 10^14^ ions/cm^2^ and 10^15^ ions/cm^2^ to simulate the effect of alpha-recoil from radionuclides in order to examine the changes to structure and disorder within variable solid solutions. It was found that macroscopic changes like swelling and loss of structural integrity generally increase in severity with increasing fluence. However, the damage appeared strongest in the solid solution members La_0.25_Ce_0.75_PO_4_ and La_0.75_Ce_0.25_PO_4_ in which it is argued that the uneven composition of the solid solution solutes, La and Ce, and their variability to displacement from ion irradiation^80^ contribute strongly to their heightened radiation intolerance. Raman mappings showed significant reductions to intensities of the ν_1_ symmetric PO_4_ stretching vibration on all examined compositions due to ion irradiation. However, both grazing incidence X-ray diffraction and HERFD-EXAFS showed that the short-range ordering was preserved within the compounds even after almost full amorphization, facilitating recrystallization. As demonstrated by GI-XRD, crystallinity could be partially restored under irradiation with 10^15^ ions/cm^2^ in all compositions. Recrystallization was found to be most effective in the end member CePO_4_, and less so in solid solution compounds and accordingly has implications for the immobilization of actinides within monazite. Overall, the study demonstrates the importance of structural chemistry of solid solution materials, the role of long- and short-range order in addition to the presence of grain boundaries in determining the bulk response and stability of a material to radiation damage.

## Data Availability

The SEM images (Figure 1), Raman spectra (Figure 2), GI-XRD measurements (Figure 4) and HERFD-EXAFS data (Figure 6) that support the findings of this study have been deposited in the data repository Zenodo under the link 10.5281/zenodo.11127328.

## Supporting Information Available

Additional experimental details, calculations, and methods, including density data, photographs of the experimental setup, further SEM images and Raman and HERFD-XANES spectra are provided in the Supporting Information file (PDF)
