# Supplementary material for: Investigation of Radiation Damage in the Monazite-Type Solid Solution La1–xCexPO4
Source: Inorg Chem. 2024 Sep 4;63(38):17525–35. doi: 10.1021/acs.inorgchem.4c02041 (PMC11423408; doi:10.1021/acs.inorgchem.4c02041)
Supplement: Supplementary file 1 — ic4c02041_si_001.pdf [file ic4c02041_si_001.pdf]

Investigation of radiation damage in the monazite-type solid solution $La_{1-x}Ce_xPO_4$

Supporting Information

Theresa Lender[1], Gabriel Murphy[2*], Elena Bazarkina[3,4], Andrey Bukaemskiy[2], Sara Gilson[3], Maximilian Henkes[2], Christoph Hennig[3,4], Alexander Kaspor[2], Julien Marquardt[5], Jonas Nießen[6], Lars Peters[1], Jenna Poonoosamy[2], André Rossberg[3,4], Volodymyr Svitlyk[3,4], Kristina O. Kvashnina[3,4], Nina Huittinen[3,7]

[1]Institute of Crystallography, RWTH Aachen University, 52066 Aachen, Germany

[2]Institute of Fusion Energy and Nuclear Waste Management (IFN-2), Forschungszentrum Jülich GmbH, 52428 Jülich, Germany

[3]Institute of Resource Ecology, Helmholtz-Zentrum Dresden-Rossendorf, 01328 Dresden, Germany

[4]The Rossendorf Beamline at ESRF, The European Synchrotron, 38043 Grenoble, France

[5]Institut für Geowissenschaften, Goethe-Universität Frankfurt, 60438 Frankfurt am Main, Germany

[6]Institut of Mineral Engineering, RWTH Aachen University, 52074 Aachen, Germany

[7]Institute of Chemistry and Biochemistry, Freie Universität Berlin, 14195 Berlin, Germany

Density measurements

Density and porosity of irradiation targets are important parameters for the interpretation of irradiation damage. Comparability between samples is only ensured if the targets are of the same quality. Green densities and porosities of the analysed samples are given in Table S1. The theoretical pellet densities were calculated by the formula: $\rho_{Th.\ pellet} = \rho_{pellet} / \rho_{Th.\ Density}$.

Table S1: Percent theoretical densities and porosities of sintered ceramics.

Composition	ρ_G, %TD	ρ_T, g/cm^3	ρ_S, g/cm^3	ρ_S, %TD	Porosity (%)	
					Open	Closed
$CePO_4$	54.9	5.1528	4.973	96.5	1.7	1.8
$La_{0.25}Ce_{0.75}PO_4$	56.9	5.1462	4.828	93.8	2	4.2
$La_{0.50}Ce_{0.5}PO_4$	56.1	5.1395	4.852	94	1.5	4.5
$La_{0.75}Ce_{0.25}PO_4$	55.5	5.1329	4.865	94.8	0.8	4.4

Penetration depth of Au ions

The penetration depth of the Au ions was determined using the Stopping and Range of Ions in Matter (SRIM) software package.[1] Default values were used for displacement threshold (25 eV for Ce, La and P; 28 eV for O), lattice binding (3 eV), surface binding energies (2-4.2 eV) and the total number of ions (99999). No significant differences were observed between the different compositions. Oxygen displacements were calculated to be dominant in all samples. The penetration depth and atomic distribution of vacancies are shown in Figure S1 for the example of $CePO_4$.

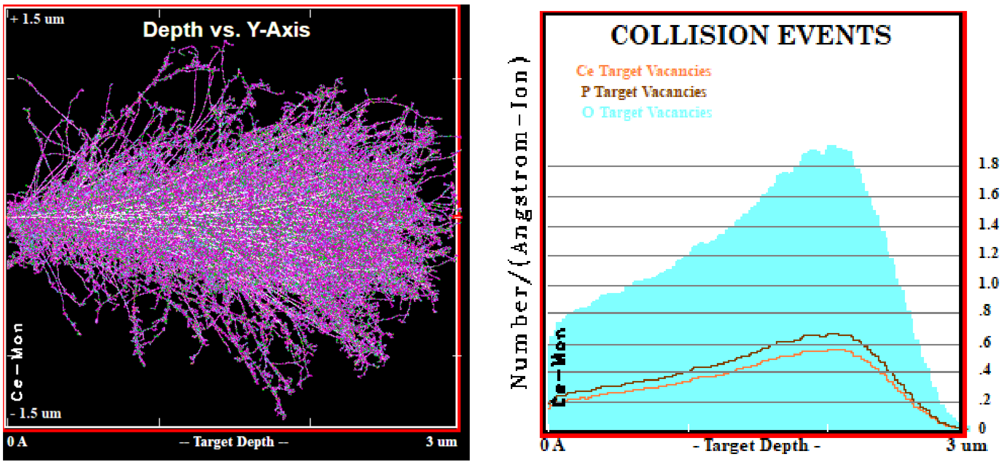

Figure S1: Full cascade calculation using SRIM, left: a penetration depth of approximately 3 µm is predicted for 14 MeV Au ions in $CePO_4$, right: vacancies are predominantly formed by displacement of oxygen atoms.

Irradiation setup

Ceramic pellets were attached to Si wafers using double-sided tape. Al foil was used to shield one half of the pellets against radiation to allow comparative studies.

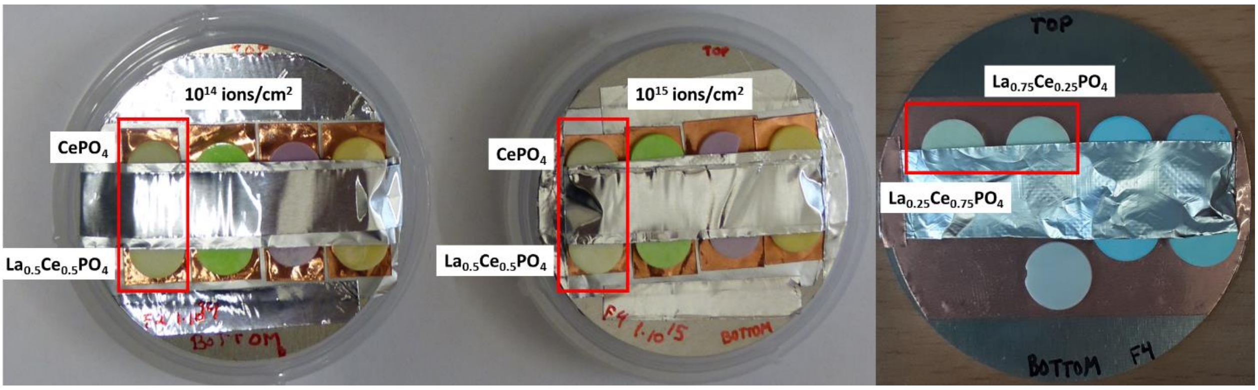

Figure S2: Image of the ceramics mounted on Si wafers for irradiation. Al foil was used for shielding.

Penetration depth of X-rays in grazing incidence mode

The angle-dependent penetration depth of X-rays was calculated with the GIXA software package.[2] The resulting graph is shown below.

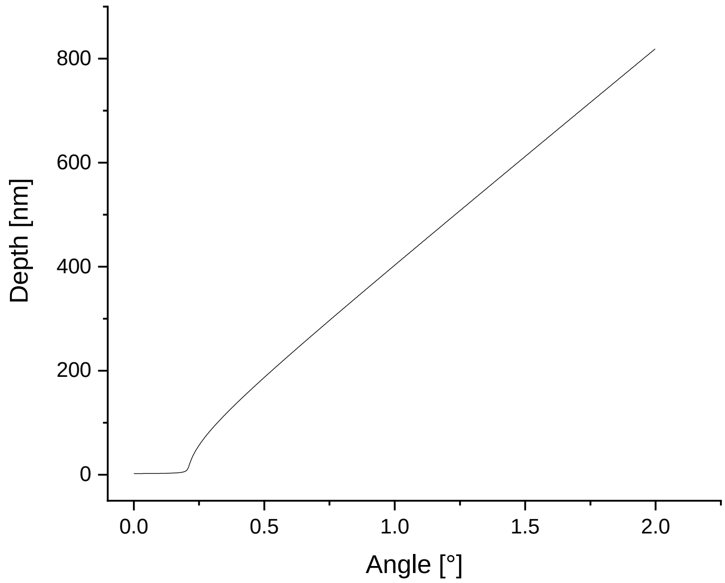

Figure S3: Penetration depth of a 12 keV X-ray beam into monazite targets.

SEM of irradiated/pristine interface

In this section, further SEM images of the interface between the irradiated and the pristine pellet areas are shown to expand on the images shown in Figure 1.

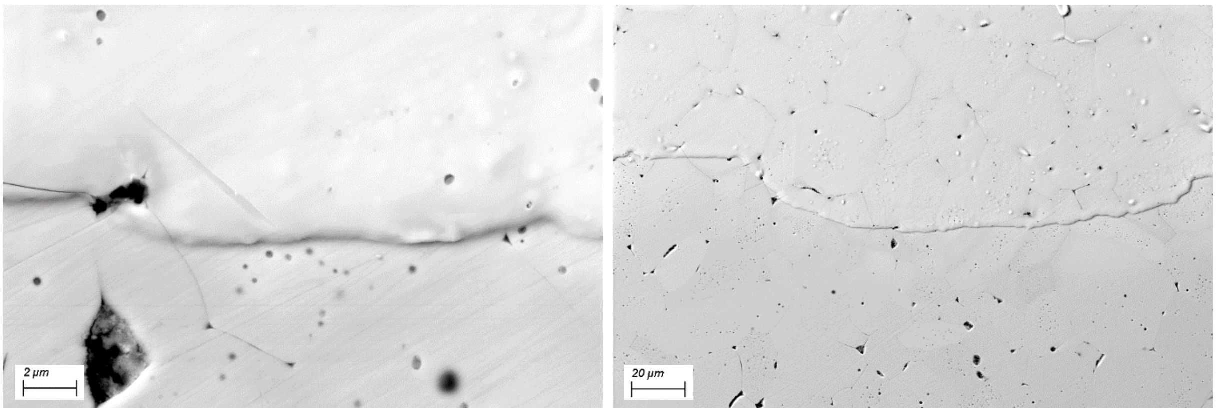

Figure S4: SEM images of the interface between irradiated (top) and pristine (bottom) $CePO_4$ irradiated with 10^{14} ions/cm^2.

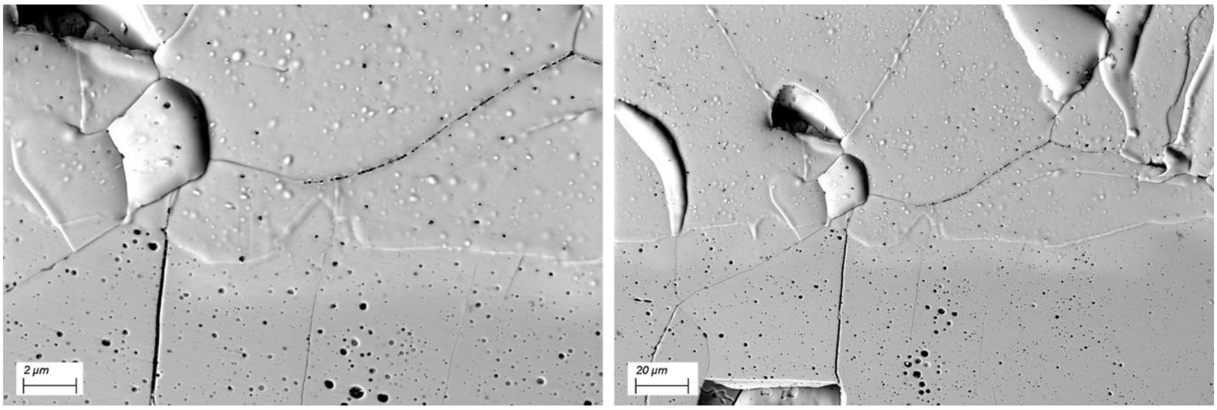

Figure S5: SEM images of the interface between irradiated (top) and pristine (bottom) $La_{0.5}Ce_{0.5}PO_4$ irradiated with 10^{14} ions/cm^2.

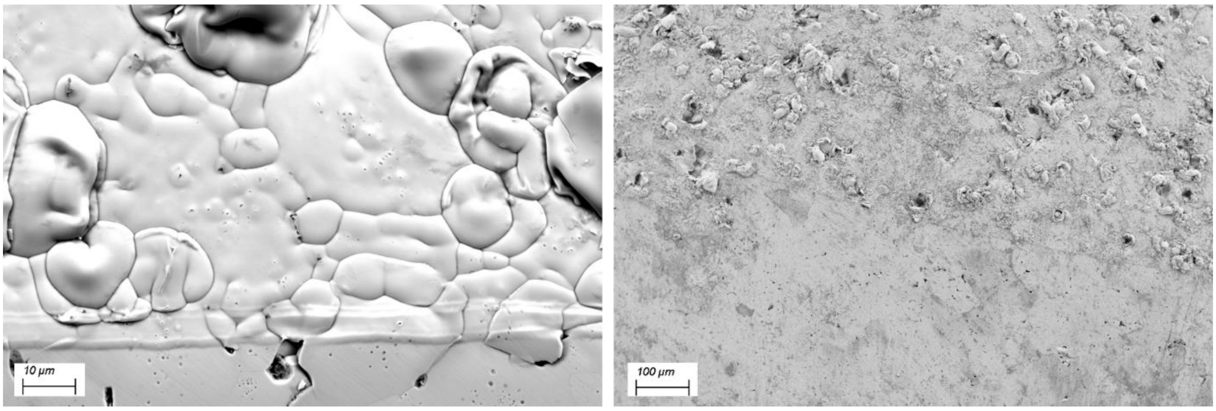

Figure S6: SEM images of the interface between irradiated (top) and pristine (bottom) $CePO_4$ irradiated with 10^{15} ions/cm^2.

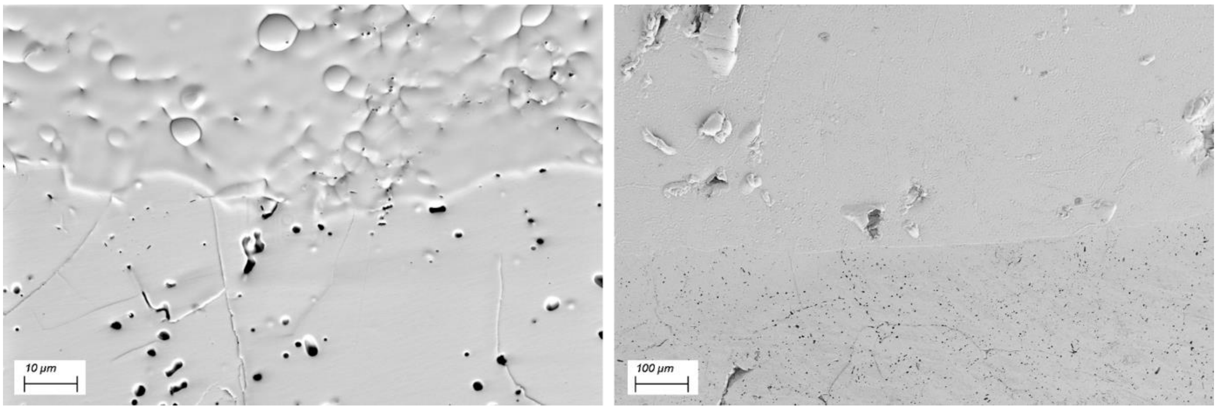

Figure S7: SEM images of the interface between irradiated (top) and pristine (bottom) $La_{0.25}Ce_{0.75}PO_4$ irradiated with 10^{15} ions/cm^2.

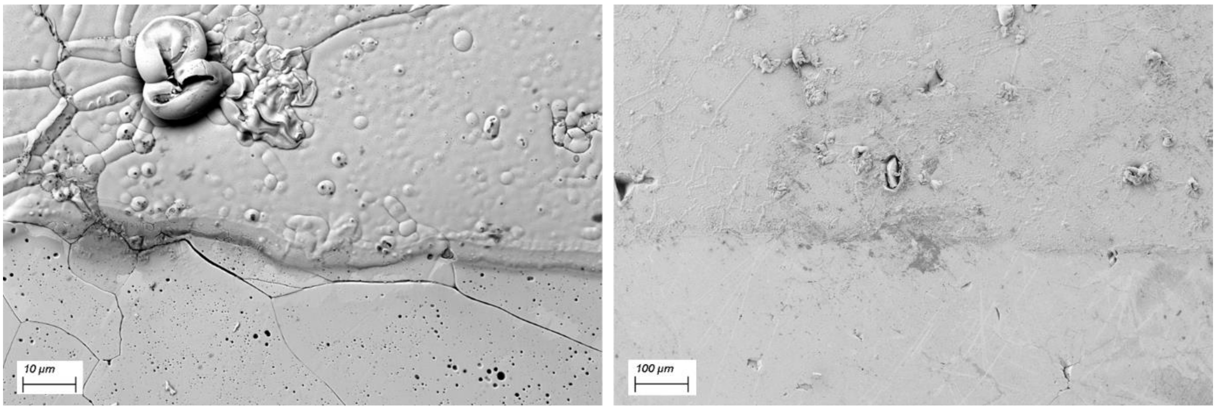

Figure S8: SEM images of the interface between irradiated (top) and pristine (bottom) $La_{0.5}Ce_{0.5}PO_4$ irradiated with 10^{15} ions/cm^2.

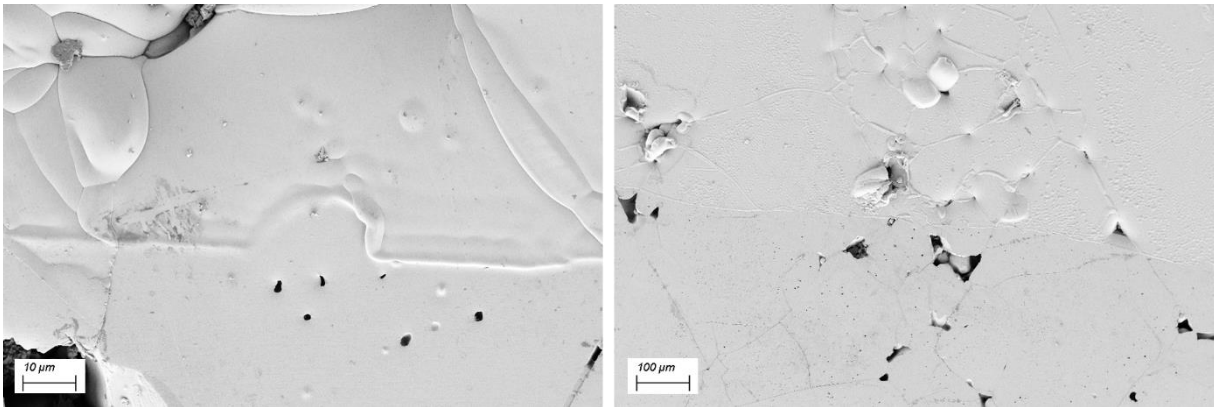

Figure S9: SEM images of the interface between irradiated (top) and pristine (bottom) $La_{0.75}Ce_{0.25}PO_4$ irradiated with 10^{15} ions/cm^2.

EDX measurements

EDX measurements were performed to confirm the chemical compositions of the irradiation targets. The results are given in Table S2.

Nominal composition	Fluence [ions/cm^2]	Atomic species	Mass fraction [at.%]
$La_{0.5}Ce_{0.5}PO_4$	10^{14}	Ce	10.1
		La	9.9
$La_{0.25}Ce_{0.75}PO_4$	10^{15}	Ce	15.0
		La	3.8
$La_{0.5}Ce_{0.5}PO_4$	10^{15}	Ce	9.6
		La	9.8
$La_{0.75}Ce_{0.25}PO_4$	10^{15}	Ce	5.1
		La	14.6

Table S2: Lanthanum and cerium content of the ceramic targets according to EDX measurements.

Raman maps

Mappings of the intensity of the v1 PO_4 stretching band are displayed in Figure S5 for all compositions. The intensity is significantly decreased in irradiated samples, resulting from the distortion of PO_4 structural units due to radiation damage. Intensity differences appear to follow the shape of crystallites, indicating that defect migration is inhibited by grain boundaries.

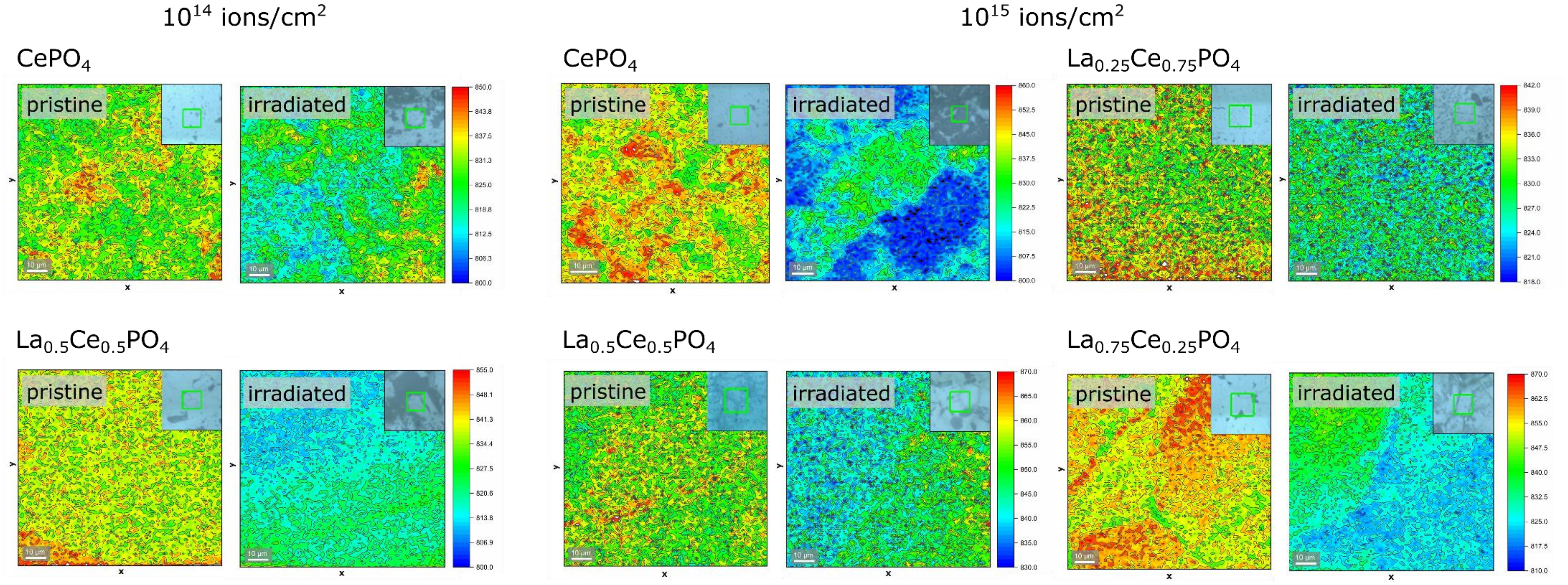

Figure S10: 2-dimensional mappings of the intensity of the v1 PO_4 stretching band measured by confocal Raman spectroscopy. Lower intensities in the irradiated areas indicate the distortion of PO_4 tetrahedra due to radiation damage. The intensity differences are more pronounced in samples irradiated with the higher fluence.

Raman spectra

Raman spectra had a rather poor signal-to-noise ratio due to short measuring times. Therefore, relevant effects can be observed more readily in mappings than by comparison of individual spectra. Spectra of $CePO_4$ irradiated with 10^{15} ions/cm^2 are shown in Figure S4. Every line is averaged from 10 individual spectra and smoothed with a 5-point Savitzky-Golay filter to improve the signal-to-noise ratio. The decreased intensity of the v1 mode in the irradiated area is clearly visible in comparison to the pristine spectrum.

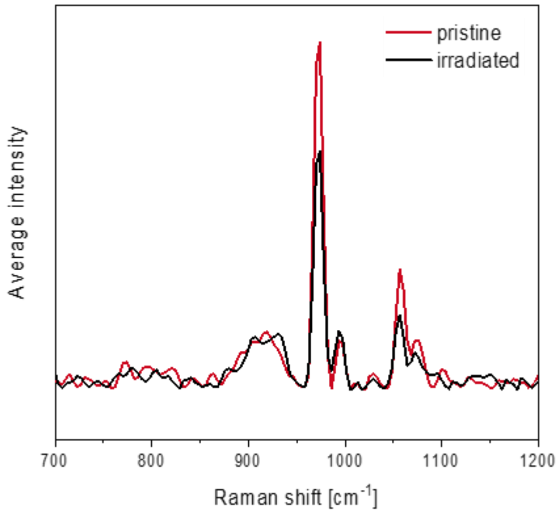

Figure S11: Raman spectra of pristine and irradiated $CePO_4$ ceramics, showing a strong decrease in the v1 mode intensity due to radiation damage in the irradiated sample.

HERFD-XANES

HERFD-XANES measurements showed that cerium was present in a trivalent state only (see Figure S5 left). A slight broadening of the white line is observed in the irradiated sample measured with an incidence angle of 45°. This broadening is more pronounced at the lower incidence angle of 9° (see Figure S5 right.) It results from the partial amorphisation of the sample.

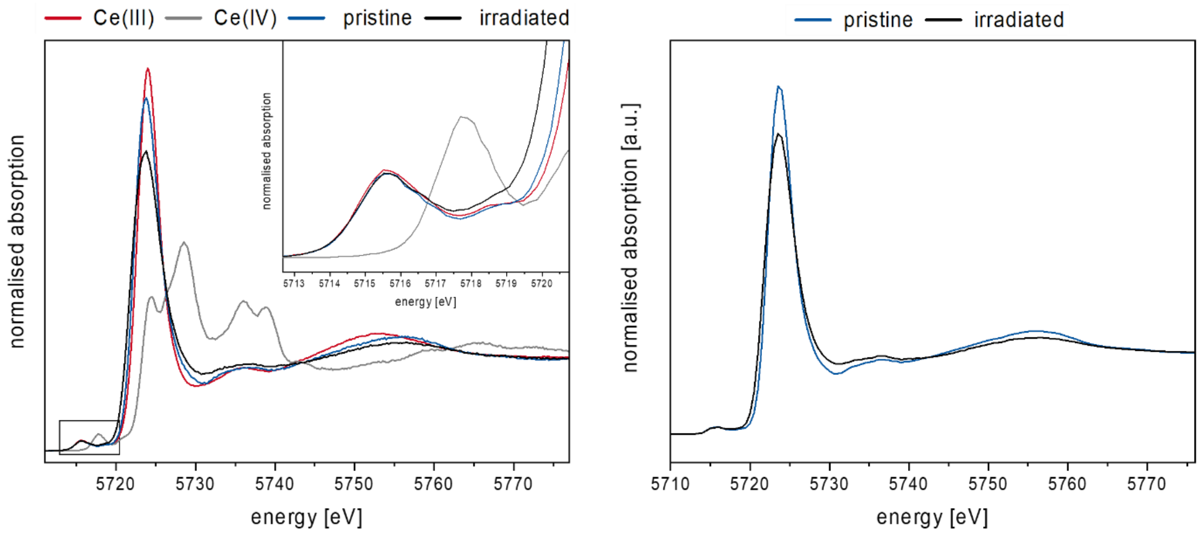

Figure S12: HERFD-XANES measurements of $CePO_4$ with an incidence angle of 45° (left) and 9° (right). The inset shows the pre-edge of $CePO_4$ compared to standards for trivalent and tetravalent cerium, proving the trivalent state of cerium in the samples. $CePO_4$ and CeO_2 were used as Ce(III) and Ce(IV) standards, respectively.

References

[1] Ziegler, J. F.; Ziegler, M. D.; Biersack, J. P. SRIM – The Stopping and Range of Ions in Matter. *Nuclear Instruments and Methods in Physics Research Section B: Beam Interactions with Materials and Atoms* 2010, *268* (11), 1818–1823. https://doi.org/10.1016/j.nimb.2010.02.091.

[2] Ingerle, D.; Pepponi, G.; Meirer, F.; Wobrauschek, P.; Streli C. JGIXA — A software package for the calculation and fitting of grazing incidence X-ray fluorescence and X-ray reflectivity data for the characterization of nanometer-layers and ultra-shallow-implants. *Spectrochimica Acta Part B: Atomic Spectroscopy* 2016, *118*, 20-28. https://doi.org/10.1016/j.sab.2016.02.010.
